# Interactive Effects of Viral and Bacterial Production on Marine Bacterial Diversity

**DOI:** 10.1371/journal.pone.0076800

**Published:** 2013-11-07

**Authors:** Chiaki Motegi, Toshi Nagata, Takeshi Miki, Markus G. Weinbauer, Louis Legendre, Fereidoun Rassoulzadegan

**Affiliations:** 1 Atmosphere and Ocean Research Institute, The University of Tokyo, Kashiwa, Chiba, Japan; 2 Center for Ecological Research, Kyoto University, Otsu, Shiga, Japan; 3 Université Pierre et Marie Curie-Paris 6, Laboratoire d'Océanographie de Villefranche, Villefranche-sur-Mer, France; 4 CNRS, Laboratoire d'Océanographie de Villefranche, Villefranche-sur-Mer, France; 5 Institute of Oceanography, National Taiwan University, Taipei, Taiwan; Universidad de Salamanca, Spain

## Abstract

A general model of species diversity predicts that the latter is maximized when productivity and disturbance are balanced. Based on this model, we hypothesized that the response of bacterial diversity to the ratio of viral to bacterial production (VP/BP) would be dome-shaped. In order to test this hypothesis, we obtained data on changes in bacterial communities (determined by terminal restriction fragment length polymorphism of 16S rRNA gene) along a wide VP/BP gradient (more than two orders of magnitude), using seawater incubations from NW Mediterranean surface waters, i.e., control and treatments with additions of phosphate, viruses, or both. In December, one dominant Operational Taxonomic Unit accounted for the major fraction of total amplified DNA in the phosphate addition treatment (75±20%, ± S.D.), but its contribution was low in the phosphate and virus addition treatment (23±19%), indicating that viruses prevented the prevalence of taxa that were competitively superior in phosphate-replete conditions. In contrast, in February, the single taxon predominance in the community was held in the phosphate addition treatment even with addition of viruses. We observed statistically robust dome-shaped response patterns of bacterial diversity to VP/BP, with significantly high bacterial diversity at intermediate VP/BP. This was consistent with our model-based hypothesis, indicating that bacterial production and viral-induced mortality interactively affect bacterial diversity in seawater.

## Introduction

Lytic viral infection accounts for a substantial fraction (10 to 50%) of bacterial mortality, and thus influences the cycling of organic carbon and nutrients in the oceans [1], [2]. In addition to their effects on biogeochemical cycling, viruses also affect bacterial community composition and diversity in marine environments [3], [4]. Because the rate of viral infection depends on the density of host cells and also because host specificity of viral infection is generally high, viruses could selectively eliminate the most dominant bacterial taxa, and thus contribute to maintain bacterial diversity [5], [6]. Consistent with this notion, some studies have shown that the presence of viruses resulted in declining abundance of the dominant group [7] and increasing diversity [8].

Other studies have found complex patterns in changes of bacterial community composition and diversity as a function of enhancement or alleviation of viral lytic pressure [9], [10], [11]. The occurrence of resistant forms of bacteria that are less susceptible to viral infection might be one factor that accounts for complex patterns in bacterial community changes, although the complexity may also stem from the fact that bacterial diversity is affected by multiple factors such as the supply of organic and inorganic nutrients supporting their growth [4], [12].

Similarly, complex responses of species diversity to changes in environmental conditions have been found in various types of ecosystems including lakes, streams, estuaries, grasslands, tropical forests and coral reefs [13], [14]. These were summarized by Kondoh [15] who reported that, on the one hand, the observed distribution of species richness as a function of disturbance (i.e., the elimination of individuals by physical and biotic forces [16]) can be either negative or positive depending on productivity, and on the other hand, the observed distribution of species richness as a function of productivity can be positive or negative depending on disturbance. Based on the dynamic equilibrium model of Huston [17], Kondoh [15] resolved this apparent paradox by modeling species richness as a function of the levels of disturbance and productivity, with the assumption that a trade-off exists between the ability for competition and the ability to resist disturbance. The model of Kondoh [15] predicted that diversity should be highest along a diagonal ridge in the parameter space of disturbance and productivity, where disturbance and productivity are balanced. In the present study, we used the general model of Kondoh [15] to test the complex responses of marine bacterial diversity to environmental changes by replacing disturbance in the original model by viral production (VP), and productivity by bacterial production (BP). Since Kondoh [15] model predicts maximum diversity at an intermediate value of the ratio disturbance/productivity, independently of the individual levels of disturbance and productivity, we tested the model prediction of a dome-shaped relationship between bacterial diversity and VP/BP. In order to generate data on changes in bacterial communities along a broad gradient of VP/BP, we collected surface water from the NW Mediterranean Sea (Bay of Villefranche) in December 2005 and February 2006, and prepared seawater incubations, i.e., non-addition control, addition of viruses-alone (V), addition of phosphate-alone (P) and addition of both phosphate and viruses (P+V). Bacterial community composition and diversity were examined by the terminal restriction fragment length polymorphism (T-RFLP) analysis of PCR-amplified 16S rRNA gene fragment [18].

## Materials and Methods

### Ethics statement

No permits were required for the field studies conducted in this work. The sampling locations were not privately-owned or protected in any way, and sampling did not involve endangered or protected species.

### Experimental set-up

Motegi et al. [1] described the methods used for water sampling, the experimental set up, the analytical procedures for bacterial and viral variables (except bacterial community analyses), and the environmental conditions at the sampling site (Point B, 43°41.00′N, 7°19.00′E, in the Bay of Villefranche, Northwestern Mediterranean). Briefly, we collected seawater samples at a depth of 10 m in the Bay of Villefranche in December 2005 and February 2006. Ninety liters of sample water were filtered through 0.8 μm pore-size filters (Isopore ATTP, diameter 142 mm; Millipore) by applying positive pressure (<67 cm Hg) using a filtration system consisting of a stainless-steel filter holder (YY3014236, Millipore), a positive pressure tank (XX6700P20, Millipore), and an air pressure pump. Particles in the <0.8 μm size fraction were concentrated (final volume, 500–780 mL) using a Pellicon filter-cassette (0.22 μm pore-size, PTGVPPC05, Millipore) to be used as ‘bacterial concentrate’. The water that passed through the 0.22 μm pore-size filters was filtered through a 100 kDa cut-off polyethersulfone membrane cartridge (Prep/scale-TFF, CDUF002TH, Millipore) to obtain a ‘viral concentrate’ (final volume, 320–480 mL) and ‘viral-free seawater’. Half of the viral concentrate was heated three times to nearly boiling temperature by microwave and chilled on ice for 10 min to be used as ‘inactivated viral concentrate’. Triplicate bottles (2–L polycarbonate, Nalgene) were prepared for each of four treatments including a non-addition control (mixture of bacterial concentrate, inactivated viral concentrate, and viral-free seawater), V treatment (mixture of bacterial concentrate, viral concentrate, and viral-free seawater), P treatment (mixture of bacterial concentrate, inactivated viral concentrate, viral-free seawater, and phosphate), and P+V treatment (mixture of bacterial concentrate, viral concentrate, viral-free seawater, and phosphate). The proportions of added bacterial concentrate, viral concentrate (or inactivated viral concentrate), and viral-free seawater were adjusted to obtain final concentrations of bacteria and viruses in each treatment that were to be equal to the in situ concentrations of bacteria and viruses at the time of sampling in the Bay of Villefranche. This adjustment of bacteria and virus concentrations was done to make the bacteria-virus interactions realistic with respect to the investigated environment. For the P and P+V treatments, NaH_2_PO_4_ was added at a final concentration of 1 μmol L^−1^. This concentration largely exceeded the phosphate concentrations in sampled waters used for the experiments (<0.01 μmol L^−1^ in both December and February), and was considered sufficient to alleviate P limitation. The bottles were incubated for 48 h at in situ temperature in the dark. Containers and plastic wares used for the sampling and preparations of incubations were rinsed before use with 1.2 N HCl followed by vigorous rinsing with Milli-Q water. Cartridge filters were cleaned with 0.1 N NaOH. During sample collection and handling, gloves were worn, and care was taken to minimize contamination. We determined bacterial (BP, μg C L^−1^ day^−1^; leucine incorporation method [19]) and viral production (VP, viruses L^−1^ day^−1^; reduction method [20]) in different treatments (Control, P, V and P+V), after 2-day incubations.

### Bacterial community analyses

The method used for bacterial community analyses was as follows. Five hundred milliliters of sample were filtered through 0.2 μm polycarbonate membrane filters (47 mm diameter; Nuclepore) and stored at −80°C until analysis. Nucleic acid extraction was performed by the cethyltrimethylammonium bromide method [21]. Ethachinmate (Wako) was used for ethanol precipitation.

PCR reactions and cycling conditions, and preparation for T-RFLP analysis were according to Moeseneder et al. [22]. The primers used for amplification by PCR of the bacterial 16S rRNA gene were the bacteria-specific primer 27F-FAM, which was 5′ end labeled with phosphoramidite fluorochrome 5-carboxyfluorescein (5′6-FAM), and the universal primer 1492R (Sigma). PCR was performed using a thermal cycler (Bio-Rad, 582BR), and each 50 μl of PCR mixture contained 5 μl of 10× *Taq* buffer, 5 μl of 2 mM dNTPs, 1 μl of Blend Taq (2.5 U μl^−1^) (Blend Taq, BTQ-101; Toyobo), 5 μl of 2 μM primers 27F-FAM and 1492R (final concentration, 0.2 μM; Sigma), 27 μl of sterilized Milli-Q water, and 2 μl of purified nucleic acid extract. PCR fragments were cleaned and concentrated using a QIAquick PCR purification kit (QIAGEN) according to the manufacturer's instructions, and were quantified using a spectrophotometer (NanoDrop, ND-1000). PCR fragments were digested using HhaI (Toyobo, HHA-101W).

Enzyme-treated and purified nucleotides were mixed with Hi-Di Formamide and a standard size maker (GeneScan, 1000 ROX, Applied Biosystems), and denatured at 90°C for 3 min. The resulting samples were loaded into a ABI Prism 3100-Avant capillary sequencer (Applied Biosystems). GeneMapper (Applied Biosystems) was used to determine the T-RFLP pattern in comparison with the internal size marker. Because background of the relative fluorescence unit (RFU) of this measurement was around ca. 8, each individual peak above 40 RFU (5 times the background) was considered to be significant, i.e., an Operational Taxonomic Unit (OTU) [10]. Duplicate samples were analyzed for each batch of amplified DNA. Only the peaks that were detected in both runs were counted, whereas the peaks that were detected in a single run were regarded as noise; in most cases, the “noise” peaks usually accounted for <5% of the total number of peaks detected according to our criterion. In the present paper, we will refer to individual OTUs by their fragment lengths, e.g., OTU 57 bp.

The number of OTU detected is OTU richness (*S*). The relative abundances of individual OTUs in a given assemblage were estimated as the relative abundance (percentage) of DNA of each individual OTU relative to the sum of the total amplified DNA. The resulting relative abundances (*P_i_*) were used to calculate bacterial diversity indices including the Shannon-Wiener diversity index (*H*), evenness (*E*), and the Simpson's index of diversity (*D*) according to the following equations (taken from Ludwig and Reynolds [23]):
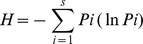


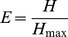


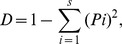
where *H*_max_ is the maximum value that *H* can reach, which corresponds to the case where all OTUs in the sample are perfectly even with one individual per OTU [*H*_max  = _ ln (S)].

### Statistical analyses

Statistical tests of the relationships between bacterial diversity indices and log VP/BP were conducted using linear (LM), quadratic (QM), cubic (CM), and additive models (AM) [24]. The AM assumes Gausian error distribution; it can divide the range of log VP/BP into multiple sections, and fit different cubic equations. There are several options in AM, but we chose the method of smoothing by cubic splines. The whole range of log VP/BP was always divided into two regions (i.e., lower and higher halves), because this provided better results (i.e., smaller Akaike Information Criterion, AIC) than division into more regions in all cases. These analyses were conducted using the R package mgcv for Generalized Linear models including Generalized Additive Models [24], [25].

## Results

### Changes in composition of dominant OTUs

**Figure 1:**
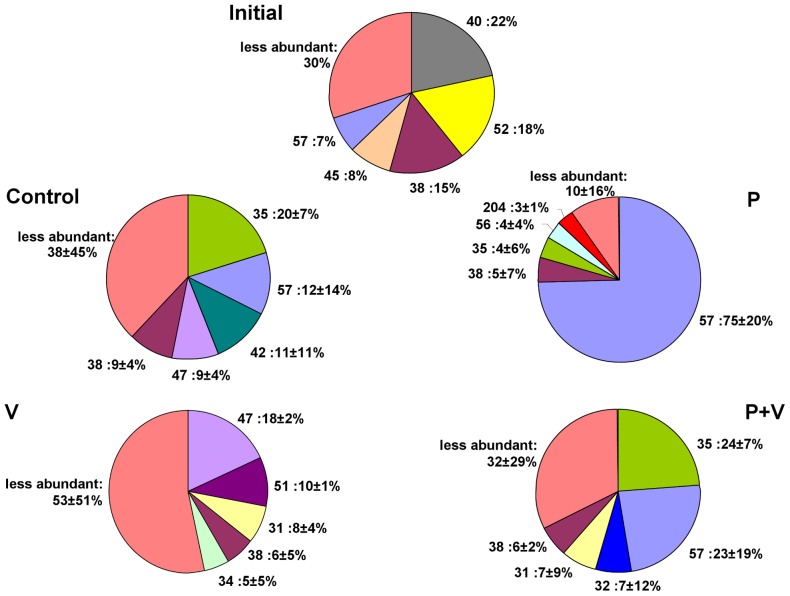
Bacterial community composition in the December experiment. Bacterial community composition at the beginning (Initial) and at the end of 2-day incubations for the four treatments: Control, virus (V), phosphate (P) and P+V, in December. The relative contributions of 5 dominant operational taxonomic units (OTUs) (indicated by code numbers) and less dominant OTUs (less abundant) are presented as percentages relative to the total amount of amplified DNA. Errors are standard deviations for triplicate bottles (n = 3) except for the seawater samples at the beginning of the incubation (n = 1).

**Figure 2:**
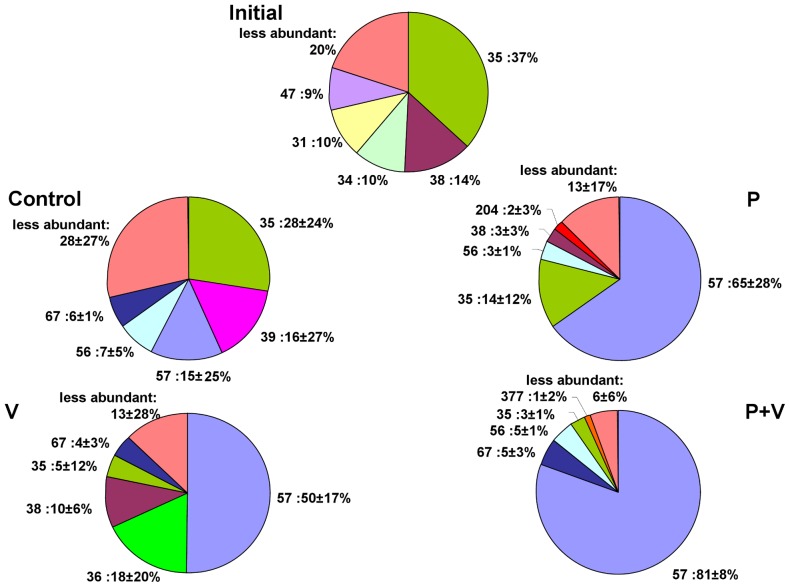
Bacterial community composition in the February experiment. Same as Figure 1 but for the February experiment.

In order to examine if the composition of the major members of bacterial communities changed in response to P and/or V additions, we estimated the relative abundances of the five dominant OTUs (denoted “dominant (s)” hereinafter), and the sum of the percentages of the remaining (less abundant) OTUs (Figs. 1 and 2). The dominants accounted for more than 70% of total amplified DNA in most cases, whereas the less abundant OTU's accounted for 6–53% of total amplified DNA.

In December (Fig. 1), three OTUs that had been dominant at the beginning of incubation (initial: 40, 52, and 45 bp) had disappeared from the list of dominants in all treatments at the end of incubation. Instead, OTU 57 bp strongly increased in abundance in the P treatment, where this OTU alone accounted for 75±20% of total amplified DNA. This was not the case in the P+V treatment, where the percentage of OTU 57 bp was significantly lower (by 3.3-fold) than that in the P treatment (p<0.05, Student's t-test). OTU 57 bp was dominant in the Control, but not in the V treatment. In February (Fig. 2), all initially dominant OTUs had disappeared or decreased considerably at the end of incubation (except for OTU 35 bp in the Control), and been largely replaced by OTU 57 bp in the P, V and P+V treatments, i.e., OTU 57 bp accounted for more than half (50–81%) of total amplified DNA in these treatments.

### Diversity of bacteria

Values of diversity indices (i.e., *S*, *E*, *H*, and *D*) in different treatments are given in Table 1. In December, there was a trend of lower diversity indices in the P treatment relative to those in the Control and V treatment, i.e., *E*, *H*, and *D* in the P treatment were significantly (ANOVA with Bonferroni corrections, p<0.05) lower than in the Control and V treatment. The reduction in diversity following P addition did not take place when viruses were added, as indicated by higher diversity indices in the P+V treatment relative to those in the P treatment; differences in *H* and *D* between these two treatments were significant (p<0.05). Diversity index *S* in the V treatment was significantly (p<0.05) higher than that in the Control and P treatment. In February, there were no significant (p>0.05) differences among treatments, with the exception of *D*, which was significantly (p<0.05) lower in the P+V treatment than in the Control and P treatment.

**Table 1:** Diversity indices of bacteria in different treatments.

Experiment	Treatment	*S*	*E*	*H*	*D*
December	Initial	17	0.84	2.39	0.88
	Control	22±7^a^	0.80±0.02^a^	2.42±0.29 ^a^	0.87±0.04 ^a^
	V	42±7^b^	0.79±0.01^a^	2.96±0.11 ^a^	0.92±0.01 ^a^
	P	9±4^a^	0.44±0.21^b^	0.97±0.62 ^b^	0.40±0.26 ^b^
	P+V	24±7^a,b^	0.70±0.05^a,b^	2.22±0.30 ^a^	0.82±0.05 ^a^
February	Initial	25	0.68	2.20	0.81
	Control	17±5^a^	0.68±0.06^a^	1.88±0.12^a^	0.73±0.04^a^
	V	11	0.64	1.47	0.65
		(7, 14)	(0.62, 0.67)	(1.30, 1.63)	(0.64, 0.67)
	P	14±12^a^	0.49±0.18^a^	1.22±0.95^a^	0.79±0.21^a^
	P+V	9±5^a^	0.39±0.06^a^	0.85±0.34^a^	0.34±0.12^b^

The letters attached to the values indicate the results of multiple comparisons (ANOVA with Bonferroni corrections) of mean diversity indices among treatments for each month (excluding the data of the V treatment in February): values with the same letter are not significantly different (p>0.05). Mean ± SD, n = 3, except the duplicate values (n = 2) presented for the data of the V treatment in February. *S*: OTU richness, *E*: Evenness, *H*: Shannon-Wiener diversity index, *D*: Simpson's index of diversity.

### Responses of bacterial diversity to VP and BP

**Figure 3:**
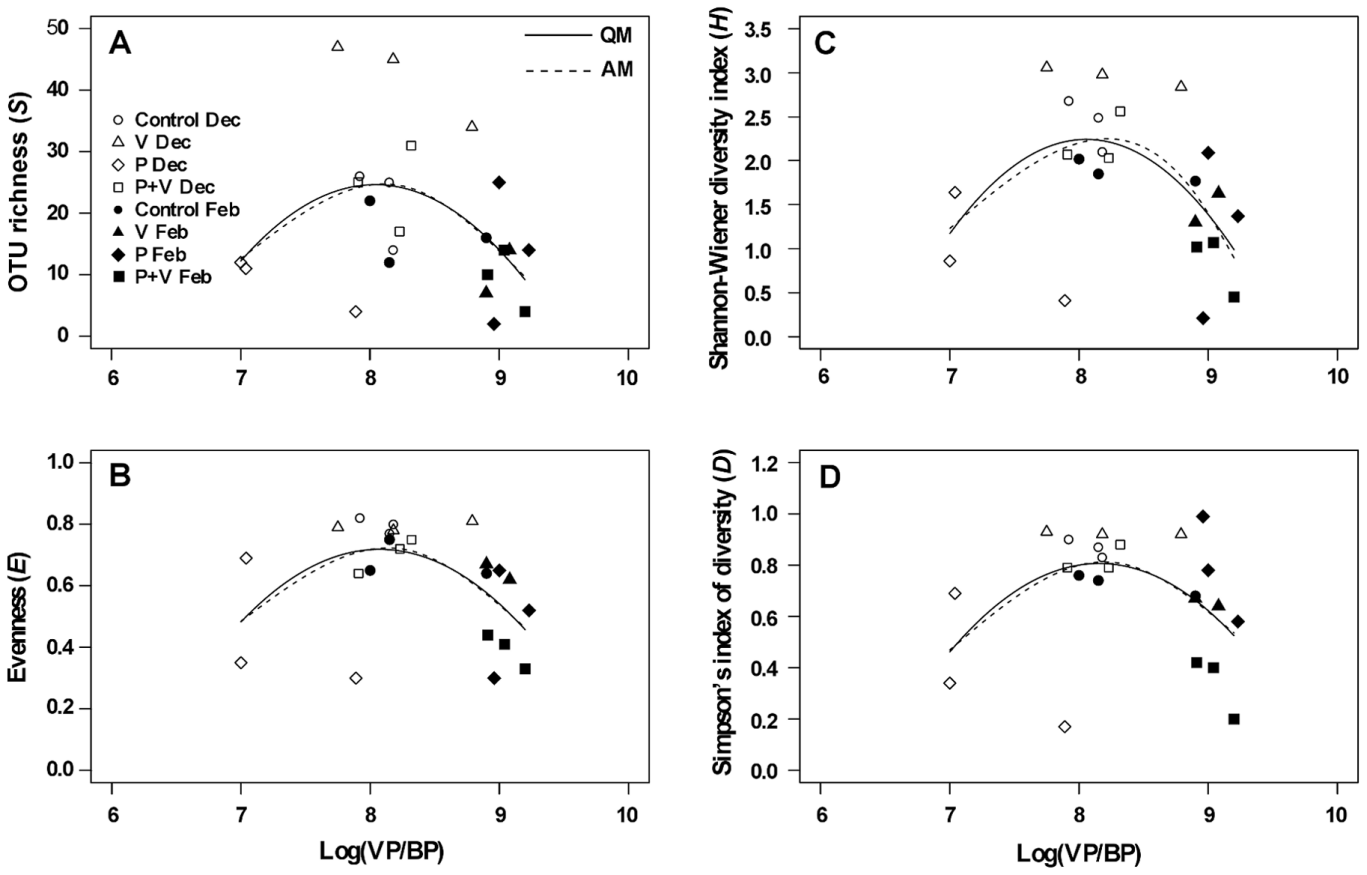
Relationships between log transformed VP/BP and four bacterial diversity indices. The figure shows the results of the relationships between log transformed VP/BP (viruses μg C^−1^) and four bacterial diversity indices (A) OTU richness, *S*, (B) evenness, *E*, (C) the Shannon-Wiener diversity index, *H*, and (D) the Simpson's index of diversity, *D*. Individual dots represent the data obtained for individual treatments in the experiments conducted in December (Control: open circle, V: open triangle, P: open diamond, P+V: open square) and February (Control: closed circle, V: closed triangle, P: closed diamond, P+V: closed square). Solid and dashed lines are quadratic regressions (QM) and additive regression (AM) of *S*, *E*, *H* and *D* on log VP/BP.

We examined the relationships between bacterial diversity indices and logarithmically transformed VP/BP (VP/BP was log transformed because it ranged over more than two orders of magnitude) using four different models: LM, QM, CM, and AM [24]. There were 8 triplicate treatments (including the 2 Controls), i.e., potentially 24 data points. For these analyses, we used the 23 available data points (one value was missing, i.e., the data of the V treatment in February) as independent realizations. The model that best explained the data (test based on AIC) for each diversity index was as follows: QM for *S* and *D*, and AM (QM was almost the same) for *E* and *H* (Table 2). Significant quadratic and/or additive regressions of *S*, *E*, *H* and *D* on log VP/BP indicated that the diversity indices were highest at intermediate log VP/BP, of which the values [95% confidence interval] were: 8.04 [3.35, 8.43], 8.07 [7.47, 8.35], 8.06 [7.44, 8.32], and 8.16 [7.52, 8.55] for *S*, *E*, *H*, and *D*, respectively (these values were calculated using QM for all diversity indices since the mgcv R package did not provide the corresponding information for AM). Because the four 95% confidence intervals overlapped, the values of log VP/BP at which the four diversity indices peaked did not differ significantly from each other. The robustness of the unimodal patterns for the four indices is attested by three results: firstly, QM was significant for all indices; secondly, AM was significant for *E* and *H*, and marginally significant for *S* and *D*; thirdly, the four diversity indices peaked within the same narrow range of log VP/BP, i.e., 7.5< log VP/BP <8.3 derived from the above 95% confidence intervals (Fig. 3).

**Table 2:** Results of statistical analyses for testing the relationship between diversity indices and log VP/BP.

Diversity index	Model^1^	Estimated parameters and/or P-value^2^	AIC
*S*	LM	NS	183.9046
	**QM**	(a, b, c) = (24.6369***, −11.4893*, 8.0401***)	**180.9766**
	CM	NS	182.8983
	AM	smooth terms are marginally significant (P = 0.0768)	181.1239
*E*	LM	NS	−10.5936
	QM	(a, b, c) = (0.71862***, −0.20543**, 8.07192***)	−16.4744
	CM	NS	−15.1924
	**AM**	**smooth terms are significant (P<0.05)**	**−16.609**
*H*	LM	NS	60.05186
	QM	(a, b, c) = (2.2435***, −0.9603**, 8.0563***)	54.04987
	CM	NS	55.95775
	**AM**	**smooth terms are significant (P<0.05)**	**54.02844**
*D*	LM	NS	3.667874
	**QM**	(a, b, c) = (0.80662***, −0.25829*, 8.15588***)	**−0.84772**
	CM	NS	−0.5264
	AM	smooth terms are marginally significant (P = 0.0576)	−0.70646

Four different models were used: linear (LM), quadratic (QM), cubic (CM), and additive (AM). Bold characters indicate the model with the lowest Akaike Information Criterion (AIC) for each diversity index. *S*: OTU richness, *E*: Evenness, *H*: Shannon-Wiener diversity index, *D*: Simpson's index of diversity.

1. Models were as follows: linear, Y∼a+ bX, quadratic, Y∼a+b (X–c)^2^, cubic, Y∼a+ bX + cX^2^ + dX^3^, additive, Y∼s (X), where X and Y are log VP/BP and one of the four diversity indices, respectively; a, b, c and d are parameters.

2. NS p>0.05, *0.01<p<0.05, **0.001<p<0.01, ***p<0.001.

## Discussion

### Methodological considerations

There are three methodological caveats to our results. Firstly, our experiments using filtered seawater did not fully capture the complexity of microbial dynamics in the sea, where the presence of organic aggregates [26] and grazers [8], [27] can strongly affect bacterial community composition and diversity. Secondly, the peak height of T-RFLP does not always reflect the numerical abundance of the corresponding OTU, because of preferential amplification, biases in DNA extraction, and differences in copy number of the rRNA operon among different bacterial OTUs [28]. Thirdly, since the T-RFLP approach using a single restriction enzyme does not provide high resolution, a single OTU discriminated by this approach (e.g., OTU 57 bp) might consist of phylogenetically diverse and ecologically distinct groups of bacteria [29]. Thus, there is a possibility that actual increases in diversity in response to changes in viral and bacterial production were underestimated in our study. Keeping these limitations in mind, the data obtained by T-RFLP fingerprinting techniques can nevertheless be interpreted as proxies of dominance and evenness (a decrease in the number of peaks indicates the dominance by fewer OTUs [9], [10]).

### Application of the general model to the analysis of bacterial diversity patterns

Our results are consistent with our initial hypothesis of a dome-shaped relationship between bacterial diversity and VP/BP. Our hypothesis was based on the model prediction of Kondoh [15] that maximum diversity should occur at a balanced ratio of disturbance to productivity independently of the individual levels of disturbance and productivity. This model unified two hypotheses that had been previously developed separately to explain how species diversity is related to disturbance and productivity, i.e., the intermediate disturbance hypothesis [13] and the productivity hypothesis [30]. Disturbance was originally defined as a physical force that excludes individuals non-selectively [13], but the concept can be extended to the mortality caused by biological agents [16]. Although biotic forces such as grazing and viral infection may exclude individuals selectively, the extent of selectivity being variable depending on the type of agent [3], [4], [31], the extension of the disturbance concept to biological agents is consistent with the fact that mortality, regardless of the kind of agent that causes it, creates opportunities for other species to acquire resources for growth, e.g., nutrients [16]. In our experiments, manipulation of viral abundance created a gradient of viral-induced mortality (i.e., disturbance) indicated by variations in VP, and addition of P led to variation in BP (i.e., productivity). Although Kondoh's model has been widely tested in various types of ecosystems (e.g., [32], [33], [34]) and in microcosms [35], our results are the first to demonstrate its applicability to the virus-bacteria system in aquatic environments. The application of the relatively simple, general model proposed by Kondoh [15] to microbial interactions brings novel perspectives and tools for investigating complex patterns of bacterial diversity in aquatic environments.

In our December experiment, the dominance of OTU 57 bp in the P treatment was substantially attenuated by the addition of viruses. Similar results have been reported in other studies, e.g., Fuhrman and Schwalbach [7] suggested that the abundance of the dominant OTU decreased with the addition of viruses in coastal seawater. These results are consistent with predictions based on the model developed by Thingstad and Lignell [5] and Thingstad [6], i.e., Killing the Winner Hypothesis. According to this model, bacteria that compete for a limited resource are eliminated by viruses in a density-dependent and species-specific manner, i.e., the model predicts that viruses will eliminate the dominant member of the bacterial community, and thus facilitate the coexistence of competitors for the same resource, hence high bacterial diversity. However, the above model prediction could not explain our February results where the abundance of the dominant taxon (OTU 57 bp) did not decrease with the addition of viruses but remained dominant (note that the actual increase in diversity could be underestimated because of the low resolution of our community analysis). One explanation for this observation could be the prevalence of lysogens that resist viral infection by homoimmunity [36], [37]. Although we lack data on proportions of lysogens in February, lysogeny has been reported to be generally common in the Mediterranean [38]. If lysogeny had been low in February, the addition of viruses would have caused increased bacterial diversity. Similarly, complex and variable patterns of bacterial diversity responses to viral-induced mortality have been reported in several studies [8], [11], [39].

Our novel application of the general model of Kondoh [15] makes it possible to explain the above complex responses of diversity to viral infection by considering the combined effects of nutrient enrichment and viral-induced mortality on bacterial diversity. The significant dome-shaped response of diversity to VP/BP supports the notion that productivity and viral induced mortality interactively affect bacterial diversity. The dome-shaped pattern mainly reflected different relationships between log VP/BP and diversity in December and February, i.e., diversity tended to decrease with decreasing log VP/BP in December, and the reverse trend was true in February. This seasonal difference would be related to differences in the effect of P addition on BP and VP between two months. In fact, the extent of BP enhancement in response to P addition was much greater in December than February (the ratio of BP in the P addition treatment relative to that in the Control was 13.2 and 1.2 in December and February, respectively [1]). In contrast, the enhancement of VP in response to P addition was more pronounced in February than in December (the ratio of VP in the P addition treatment relative to that in the Control was not significantly different from unity in December whereas the ratio was 5.3 in February [1]). The overall consequence was that P addition resulted in high (as high as 9.2) and low (as low as 7.0) log VP/BP in February and December, respectively. The observed differential responses of BP and VP (and VP/BP) to P addition could have been caused by differences in the extent of P limitation, or concentration and composition of dissolved organic substrates. Alternatively, the seasonal difference could have been caused by a difference in species sources in the sample waters used for the experiments. However, it is generally considered that bacterial species sources differ little in coastal waters (“everything is everywhere”, [40]) because of extensive mixing that facilitates immigration of bacterial taxa [41], even though community compositions of the major taxa, as evidenced by 16S rDNA fingerprinting, may display large spatio-temporal variability (e.g., [42]). We consider that the seasonal shift in the relationship between diversity and log VP/BP was caused by seasonal differences in responses of bacteria and viruses to P addition and not to the presence or absence of particular species in the original waters, although rigorous testing of this assertion would require further investigations in which the bacterial community would be analyzed using high resolution sequencing techniques (e.g., [43]).

Our approach strikingly differs from previous studies in which effects of nutrient enrichment and viral-induced mortality on marine bacterial diversity had been examined separately (reviewed by Winter et al. [4]). The unifying approach that we used here is simple, i.e., the formulation uses only two parameters, VP and BP, that are easy to determine. Although the dome-shaped pattern was observed presumably due to a wide VP/BP gradient, this approach is also readily applicable to field settings by testing the null hypothesis that the data are randomly distributed relative to BP and VP through regression of bacterial diversity indices on VP/BP. Our compilation of VP/BP data collected in the ocean (Table S1) shows that log VP/BP displays wide variability (range, 7 to 11) depending on depth and geographical region, although variability in VP/BP could be partly due to differences in methods for determining VP and BP. Future studies should examine the mechanisms that generate wide VP/BP ranges in oceans, and test the prediction that bacterial diversity is maximum when log VP/BP has an intermediate value (which was around 8 in the present study). In marine waters where protist grazers are important agents of bacterial mortality, the model framework could be modified by adding the protist-induced bacterial mortality to VP.

Generally in ecology, the trade-off between superiority in competition and resistance to disturbance provides a theoretical explanation for diversity patterns along a productivity-disturbance gradient [15], [17]. For bacteria-virus systems in aquatic environments, some studies have suggested that a trade-off indeed exists between the bacterial traits of enhanced nutrient uptake (superiority in competition) and those of reduced mortality caused by viruses (resistance to disturbance) [12], [39], [44]. Thingstad et al. [12] suggested that the increase in the number of nutrient transporters in membrane is advantageous for nutrient competition but disadvantageous for avoidance of viral infection. Other studies indicated that the fastest growing bacterial group (superior in nutrient competition) was the most susceptible to viral-induced mortality, suggesting the existence of a trade-off between nutrient uptake and resistance to viral infection [4], [39]. However, the key assumption of the trade-off model that each trait should be accompanied by its own fitness cost has yet to be fully validated, and one study has argued that the cost for the resistance to viral infection would be low, if not negligible, in cyanobacteria [45]. Kadmon and Benjamini [46] proposed that the dome-shaped pattern of diversity along the productivity-disturbance gradient could be explained by a simple neutral model with no assumption of trade-offs between competitive ability and tolerance to disturbance. Elucidation of the mechanisms and processes involved in the dome-shaped response of bacterial diversity to the VP/BP gradient that we found in the present study could be the aim of future studies.

## Supporting Information

Table S1 **Values of log VP/BP in various regions of the ocean, together with information on the methods used for determining BP and VP.** Units of VP and BP are viruses L^−1^ day^−1^ and μg C L^−1^ day^−1^, respectively. BP reported in the source literature was converted to the unit of μg C L^−1^ day^−1^, using a conversion factor (or conversion factors), if necessary (see the footnotes).
(DOC)
