# Supplementary material for: Interactive Effects of Viral and Bacterial Production on Marine Bacterial Diversity
Source: PLoS One. 2013 Nov 7;8(11):e76800. doi: 10.1371/journal.pone.0076800 (PMC3820650; doi:10.1371/journal.pone.0076800)
Supplement: Table S1 — Values of log VP/BP in various regions of the ocean, together with information on the methods used for determining BP and VP. Units of VP and BP are viruses L−1 day−1 and μg C L−1 day−1, respectively. BP reported in the source literature was converted to the unit of μg C L−1 day−1, using a conversion factor (or conversion factors), if necessary (see the footnotes). (DOC) [file pone.0076800.s001.doc]

Table S1. **Values of log VP/BP in various regions of the ocean, together with information on the methods used for determining BP and VP.** Units of VP and BP are viruses L^-1^ day^-1^ and μg C L^-1^ day^-1^, respectively. BP reported in the source literature was converted to the unit of μg C L^-1^ day^-1^, using a conversion factor (or conversion factors), if necessary (see the footnotes).

| Region | Log VP/BP (range) | Determination methods**^a^** | | References |
| --- | --- | --- | --- | --- |
|  |  | BP | VP |  |
| Northwestern Mediterranean (Blanes Bay) | 7.6- 8.2**^b^** | ΔBA | ΔVA | Boras et al. (2009) |
| Mediterranean (North Adriatic) | 10.0-10.6 | Leu | ΔVA | Bongiorni et al. (2005) |
| Atlantic | 7.2-10.0**^b^** | ΔBA | ΔVA | De Corte et al. (2012) |
| Central South Pacific | 8.5-10.1**^b,c^** | TdR | TdR | Motegi and Nagata (2007) |
| Western North Pacific | 9.1-10.9 | Leu | TdR | Motegi et al. (2009) |
| Western North Pacific (Otsuchi Bay) | 8.6- 8.9**^b^** | TdR | TdR | Motegi and Nagata (2009) |
| Australian Southern Ocean | 9.1- 9.7 | ΔBA | ΔVA | Evans et al. (2009) |

1. TdR: ^3^H-thymidine method, Leu: ^3^H-leucine method, ΔBA: BP determined from changes in bacterial abundance (losses corrected depending on studies), ΔVA: VP determined from changes in viral abundance (viral abundance was reduced at the beginning of the incubation).
2. A conversion factor of 20 fg C cell^-1^ was used to adjust the BP unit.
3. A conversion factor of 2.2 x 10^18^ cells mole TdR^-1^ was used to adjust the BP unit.

**Reference**

Bongiorni L, Magagnini M, Armeni M, Noble R, Danovaro R (2005) Viral production, decay rates, and life strategies along a trophic gradient in the North Adriatic Sea. Appl Environ Microbiol 71:6644-6650.

Boras JA, Sala MM, Vázquez-Domínguez E, Weinbauer MG, Vaqué D (2009) Annual changes of bacterial mortality due to viruses and protists in an oligotrophic coastal environment (NW Mediterranean). Environ Microbiol 11: 1181–1193.

De Corte D, Sintes E, Yokokawa T, Reinthaler T, Herndl GJ (2012) Links between viruses and prokaryotes throughout the water column along a North Atlantic latitudinal transect. ISME J 6: 1566–1577.

Evans C, Pearce I, Brussaard CP (2009) Viral-mediated lysis of microbes and carbon release in the sub-Antarctic and Polar Frontal zones of the Australian Southern Ocean. Environ Microbiol 11: 2924–2934.

Motegi C, Nagata T (2007) Enhancement of viral production by addition of nitrogen or nitrogen plus carbon in subtropical surface waters of the South Pacific. Aquat Microb Ecol 48:27-34.

Motegi C, Nagata T (2009) Addition of monomeric and polymeric organic substrates alleviates viral lytic pressure on bacterial communities in coastal seawaters. Aquat Microb Ecol 57:343-350.

Motegi C, Nagata T, Miki T, Weinbauer MG, Legendre L, et al. (2009) Viral control of bacterial growth efficiency in marine pelagic environments. Limnol Oceanogr 54:1901-1910.
